# Interaction with the entomopathogenic fungus *Beauveria bassiana* influences tomato phenome and promotes resistance to *Botrytis cinerea* infection

**DOI:** 10.3389/fpls.2023.1309747

**Published:** 2023-12-19

**Authors:** Assunta Russo, Jana Barbro Winkler, Andrea Ghirardo, Maurilia M. Monti, Susanna Pollastri, Michelina Ruocco, Jörg-Peter Schnitzler, Francesco Loreto

**Affiliations:** ^1^ University of Naples Federico II, Department of Agricultural Sciences, Portici, Italy; ^2^ National Research Council of Italy, Institute for Sustainable Plant Protection (CNR-IPSP), Portici, Italy; ^3^ Helmholtz Zentrum München, Research Unit Environmental Simulation, Neuherberg, Germany; ^4^ Department of Biology, University of Naples Federico II, Naples, Italy

**Keywords:** beneficial microorganisms, photosynthesis, plant pathogens, plant phenotyping, volatile organic compounds

## Abstract

Plants are central to complex networks of multitrophic interactions. Increasing evidence suggests that beneficial microorganisms (BMs) may be used as plant biostimulants and pest biocontrol agents. We investigated whether tomato (*Solanum lycopersicum*) plants are thoroughly colonized by the endophytic and entomopathogenic fungus *Beauveria bassiana*, and how such colonization affects physiological parameters and the phenotype of plants grown under unstressed conditions or exposed to the pathogenic fungus *Botrytis cinerea*. As a positive control, a strain of the well-known biocontrol agent and growth inducer *Trichoderma afroharzianum* was used. As multitrophic interactions are often driven by (or have consequences on) volatile organic compounds (VOCs) released by plants constitutively or after induction by abiotic or biotic stresses, VOC emissions were also studied. Both *B. bassiana* and *T. afroharzianum* induced a significant but transient (one to two-day-long) reduction of stomatal conductance, which may indicate rapid activation of defensive (rejection) responses, but also limited photosynthesis. At later stages, our results demonstrated a successful and complete plant colonization by *B. bassiana*, which induced higher photosynthesis and lower respiration rates, improved growth of roots, stems, leaves, earlier flowering, higher number of fruits and yield in tomato plants. *Beauveria bassiana* also helped tomato plants fight *B. cinerea*, whose symptoms in leaves were almost entirely relieved with respect to control plants. Less VOCs were emitted when plants were colonized by *B. bassiana* or infected by *B. cinerea*, alone or in combination, suggesting no activation of VOC-dependent defensive mechanisms in response to both fungi.

## 1 Introduction

The use of beneficial microorganisms (BMs) has been promoted in recent years as a novel strategy to ensure food safety and security of agricultural products while reducing the application of pesticides and chemical fertilizers and pursuing agroecology principles (Parnell et al., 2016). In this context, plant protection by endophytic fungi may be also considered, establishing a mutually beneficial symbiotic relationship with the host plant and being exploited as an alternative source of secondary metabolites (Bamisile et al., 2018; Sinno et al., 2020; Wen et al., 2022). The entomopathogenic fungus, *Beauveria bassiana* (Bals.) Vuill. (Ascomycota: Hypocreales) can endophytically colonize tissues of many plants. Colonization was successfully demonstrated by using different inoculation methods such as seed coating, soil watering, root dipping, and foliar spraying (Tefera and Vidal, 2009). *Beauveria bassiana* is well-known for its endophytic potential in the biocontrol of insect herbivores (Vega, 2018), and its mechanism of action as an entomopathogen has been extensively studied (Ortiz-Urquiza and Keyhani, 2013; Pedrini et al., 2013; Wang et al., 2021). More recently, *B. bassiana* was proposed as a dual-purpose microbial control organism against both insect pests and plant pathogens (Allegrucci et al., 2017; Saranraj and Jayaparakash, 2017; Wei et al., 2020). In addition, tomato plants colonized by *B. bassiana* apparently show improved nutrient root uptake, perhaps via enhanced activity of phytohormones or growth regulators (González-Guzmán et al., 2022).

There are several bioformulates based on *B. bassiana* that are already commercially available, and interest on the practical use of this and other BMs is spurring further research (Felizatti et al., 2021). However, despite some efforts (Macuphe et al., 2021), the effect of *B. bassiana* on plant physiology and improvement of plant resistance to pathogens has just recently started to be investigated (Proietti et al., 2023).

To evaluate whether endophytic colonization by *B. bassiana* strain ATCC 74040 (Naturalis, CBC Europe s.r.l.,Biogard division, Grassobbio, Italy) affects the plant phenome, we used tomato (*Solanum lycopersicum*) plants. In particular, we studied whether *B. bassiana* colonization a) expands quickly across plant vegetative and reproductive organs; b) is quickly sensed by plants, causing the onset of defensive responses; c) has a biostimulant effect inducing long-term changes in the plant phenotype; d) improves plant resistance to *Botrytis cinerea* (the gray mold), a destructive fungal pathogen of a wide range of fruits, vegetable and ornamental crops, and considered a “high-risk” necrotrophic pathogen characterized by short life cycle, high reproduction, and large genetic variation (Poveda et al., 2020). Our results prompt for a rapid and complete plant colonization of *B. bassiana* that is first recognized as a foreign invader, and then rapidly elicits plant growth and protection against the pathogen *B. cinerea*.

## 2 Materials and methods

### 2.1 Experimental protocol, plant material and growth conditions

Experiments were performed for two years in two different research institutes. In the first year, the impact of *B. bassiana* colonization on plant physiology (primary metabolism) and phenotype was assessed at the facilities of the National Research Council of Italy (CNR-IPSP) in Portici (Naples, Italy). *Trichoderma afroharzianum* (strain T22), largely used as biocontrol agent (BCA) and plant growth promoter (Thapa et al., 2020), was used as a benchmark. The second year, the experiment was carried out at the Research Unit Environmental Simulation (EUS), Helmholtz Zentrum München (HMGU, Munich, Germany), where we concentrated on measuring the effect of *B. bassiana* on tomato plants for a longer time course (up to fruiting) and also followed whole plant phenotyping, VOC emission, and impact of *B. bassiana* on a subsequent infection by *B. cinerea.*

Tomato seeds (*Solanum lycopersicum* cv San Marzano nano, Semiortosementi, Sarno, Italy) were surface-sterilized in 1% NaOCl (v/v) for 5 min, rinsed twice with sterile distilled water (SDW) and germinated on Whatman sterile filter paper (Sigma-Aldrich, Darmstadt, Germany) soaked with SDW, in the dark, at 24°C. Germination occurred in 4-5 d. Seedlings were firstly individually transplanted to 8 cm diameter pots (1.3 L) of non-sterile commercial soil (Universal potting soil-Floragard Vertriebs-GmbH Oldenburg), then potted in 13 cm diameter pots (2.16 L) and kept in growth chambers (Italy) or a climatized greenhouse (Germany) at 25 ± 2°C, 70 ± 10% RH, and a photoperiod of 14:10 h (light:dark), with a photosynthetic active radiation (PAR) of around 700 mmol m^-2^ s^-1^ during the days. More than 200 plants were grown to conduct all following experiments.

### 2.2 Fungal cultures

*Beauveria bassiana* strain ATCC 74040 (Naturalis), *B. cinerea* (isolate B05.10) and *T. afroharzianum* (strain T22) were cultured on 4.5 g 100 mL^-1^ Potato Dextrose Agar (PDA from Sigma-Aldrich, St. Louis, MO, USA), maintained at 25 ± 2°C, and 14:10 h (light:dark) photoperiod for 20 d. For conidial production, aerial conidia from all the fungi were harvested by flooding the plate with sterile distilled H_2_O containing 0.02% Tween 80 (Sigma-Aldrich). Conidial suspensions were filtered with a sterile pipette tip plugged with cotton wool and final conidial concentrations were determined by direct count using a haemocytometer (Neubauer hemocytometer chamber) under a microscope and adjusted to the indicated concentration for final use.

### 2.3 Induction and assessment of endophytic colonization by *Beauveria bassiana*

Emerged tomato seedlings (27-d old plants) were treated by drenching soil with 50 mL of 1 × 10^6^ conidia mL^-1^ of a conidial solution of *B. bassiana*. Control plants were watered with the same volume of SDW. The same treatment was repeated after a week on 35-d old plants. From this second treatment with *B. bassiana*, we counted days post inoculum (dpi) for all the experiments.

To confirm *B. bassiana* endophytic colonization, tissue samples were collected from leaves of 5 treated and 5 control tomato plants at 1, 2, 7, 15, 21, 35, 42, 49, 56, 63 dpi, respectively. At the fruiting time, 5 tomato fruits from treated and control plants were also harvested, and tomato seeds were collected. Leaf samples and tomato seeds were randomly chosen, and surface sterilized in 1% NaOCl for 3 min, after which they were rinsed 3 times with SDW. These steps were useful to ensure that appearance of mycelial growth was only due to *B. bassiana* growing inside plant tissues. The success of the disinfection procedure was assessed by plating three replicates of 100 µL each of the residual rinsed water on PDA-medium plates. Leaf samples and seeds were dried on sterile paper, leaves were cut into pieces of about 1 cm^2^ each and seeds into half; both leaves and seeds were placed on 90 mm wide Petri plates containing PDA supplied with 1% (v/v) lactic acid to avoid bacterial contamination. Plates were incubated at 25°C in the dark. Leaf pieces and seeds were monitored daily to determine if there was fungal growth emerging from the cut plant tissues (white, cottony, dense hyphal growth of *B. bassiana*) (Humber, 2012). The fungal mycelia were isolated and transferred on new plates containing PDA, in order to obtain pure cultures for their morphological identification.

### 2.4 *Botrytis cinerea* infection and evaluation of damage in tomato plants

Four days after the second treatment in soil with *B. bassiana*, a conidial solution of the pathogenic fungus *B. cinerea* (20 ml of 1×10^5^ conidia mL^-1^) was applied as a foliar spray on each 39-d old tomato plant. The plants were maintained in water-sprayed boxes for 4 d to ensure high humidity favouring conidial germination. Control plants received the same spraying treatment but with SDW. The disease development was evaluated at 9, 10, 12, 15 dpi by calculating the ratio between the surface area affected by the pathogen and the total leaf area. These two areas (cm^2^) were estimated by harvesting and scanning leaves and stems. The digital images obtained were analysed with an open-source image processing software (Jud et al., 2018). All pixels of the image representing plant leaves were calculated and interpolated with leaf area values using a cubic spline function. The software allows to manipulate and adjust colour threshold parameters (hue, saturation, and brightness value), quantitatively determining the total leaf area (hue= 17/180, saturation =15/100 and brightness value=18/100) and the leaf area covered by sporulation or damaged, with necrotic and chlorotic symptoms) (hue= 4/61, saturation= 11/51 and brightness value= 7/100). Since the first stage, *B. cinerea* infection causes yellowing of leaves, which is a rather unspecific symptom. Indeed, leaf necroses in plants without *B. cinerea* infection (i.e. treated with *B. bassiana* and in control conditions) are also reported, and were considered in our experiment (added to background) to help differentiate unspecific necrosis from symptoms due to pathogen infection. This experiment was preceded by a pilot experiment to set up the best timing for disease development detection.

### 2.5 Phenotyping of control and *Beauveria bassiana*-colonized plants: growth

Five tomato plants for each treatment were randomly chosen, uprooted, and dissected at root, stem and leaf level at 1, 2, 7, 15, 21, 35, 42, 49, 56, 63 dpi, to analyse plant growth data, respectively. Roots, stems and leaves fresh weights (FW) were determined. Roots were carefully washed under tap water to remove the soil. Dry weights (DW) of roots, stems and leaves were obtained after drying samples in an oven at 70°C for 72 h. Root length, stem height, number of flowers and fruits, and tomato fruit weights were also measured.

### 2.6 Phenotyping of control and *Beauveria bassiana*-colonized plants: gas-exchange, chlorophyll fluorescence, and emission of volatile organic compounds

#### 2.6.1 Gas-exchange and chlorophyll fluorescence at leaf level

Measurements of gas exchange (CO_2_ and H_2_O) and chlorophyll fluorescence were conducted by enclosing fully mature leaves in an 8-cm^2^ leaf cuvette surface of an Infra-Red Gas Analyzer system [standard measuring head 3010-S of a portable system for simultaneous analysis of gas exchanges and chlorophyll fluorescence GFS-3000 (Heinz Walz GmbH, Effeltrich, Germany)]. All measurements were conducted between 8:00 am and 3:00 pm. After a dark adaptation period of at least 30 min, the leaf was illuminated under standard conditions (PAR 1000 µmol m^−2^ s ^−1^, leaf temperature 30°C, relative air humidity 50%, and CO_2_ concentration set to 400 ppm, matching ambient CO_2_ levels) until stomata opened and steady state CO_2_ and water vapour exchange rates were reached. Values of net photosynthesis, aka CO_2_ assimilation (Pn), transpiration (Tr), stomatal conductance to water vapor (gH_2_O), intercellular CO_2_ concentration (Ci) and respiration in the dark (Rd) were calculated from gas-exchange measurements (Von Caemmerer and Farquhar, 1981; Farquhar and Sharkey, 1982). Minimum fluorescence (Fo), maximal fluorescence in the dark-adapted leaf (Fm) or light-adapted leaf (Fm′), steady state fluorescence in the light-adapted leaf (Fs), and minimal fluorescence in the light-adapted leaf (Fo′) were determined, as described previously (Maxwell and Johnson, 2000). The maximal quantum yield of PSII was calculated as: Fv/Fm = (Fm – Fo)/Fm, while the effective quantum yield of PSII in illuminated leaves was calculated as: ΦPSII = (Fm′ – Fs)/Fm′ (Genty et al., 1989). The electron transport rate was calculated by multiplying the ΦPSII with the amount of PAR absorbed by PSII: ETR = (ΦPSII) × (PAR) × (0.84) × (0.5), where 0.84 and 0.5 estimate that leaves absorb 84% of incident photons, 50% of which are absorbed by PSII, assuming that the absorbed light is equally distributed between photosystem I and II (Yamori et al., 2011). Non-photochemical energy quenching (NPQ), a measure of heat dissipation of absorbed light energy, was calculated as: NPQ = (Fm/Fm′) - 1, while Y(NPQ) which is the fraction of PAR that is dissipated in PSII via the non-photochemical quenching mechanisms was calculated as Y(NPQ) = F/Fm′ − F/Fm (Bilger and Björkman, 1990; Maxwell and Johnson, 2000).

#### 2.6.2 Gas-exchange and VOCs at entire plant level

**Figure 1:**
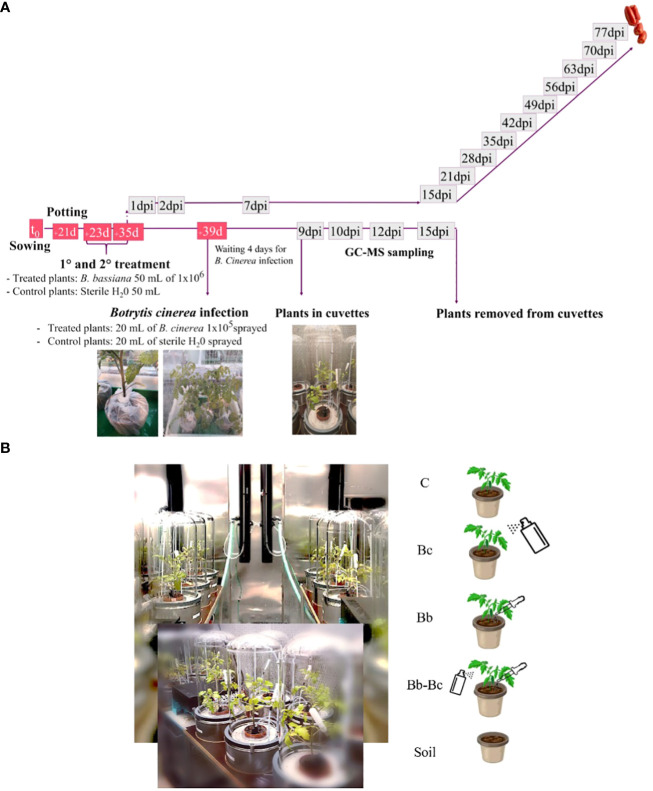
Experimental workflow is represented in figure **(A)**; after sowing and potting, plants were inoculated twice with *Beauveria bassiana*, provided in the irrigation water. Controls were irrigated only with water. Phenotyping of growth parameters, gas-exchange and chlorophyll fluorescence was carried out in treated and control plants from one day post inoculum (dpi) until fruiting. For experiment in the VOC-SCREEN platform, 39d-old plants that were previously either inoculated by *B. bassiana* or kept in control conditions, were infected with *Botrytis cinerea* and gas exchange was measured in cuvettes until 15 dpi **(B)**. Cuvettes in the VOC-SCREEN platform were divided according to different plant treatments: control plants (C); *B. cinerea* spray-infected plants (Bc); *B. bassiana*-colonized plants (Bb); Plants colonized by *B. bassiana* and sprayed with *B. cinerea* (Bb-Bc); soil pots only (soil).

Gas exchange and VOC measurements were conducted in the VOC-SCREEN platform (Jud et al., 2018), installed in one of the phytotron chambers at HMGU, where it was possible to control environmental parameters such as temperature, relative humidity (RH), photosynthetically active radiation (PAR), maintaining them similar to growth conditions (see above). The cuvettes in which the plant pots were installed (total volume of ∼ 40 L) were made of stainless steel and a cylindrical Duran glass cover clamped to the base with inert Viton rings sealing the joint. The base of the cuvette contained gas and irrigation tubing and electrical connections. From the cuvette base, the supplied air was flushed into the cuvette air space via a circular system of dozens small inlet holes (Jud et al., 2018). Following the experimental setup of **Figure 1A**, plants were incubated for 4 d to make possible *B. cinerea* conidial germination before being enclosed in the cuvettes (9 dpi) and kept under observation for 1 week. At 16 dpi, plants were removed from cuvettes and discarded. The 24 cuvettes of the platform were divided according to the following experimental design, as shown in **Figure 1B**: 5 control (C) cuvettes hosted a potted tomato plant each, grown without any fungal treatment; 5 cuvettes (Bb) hosted a potted tomato plant each, where *B. bassiana* spores were inoculated by soil irrigation in two treatments (as described in section 2.3); 5 cuvettes (Bc) hosted a potted tomato plant each, where the pathogen *B. cinerea* was sprayed (as described earlier); 5 cuvettes (Bb-Bc) hosted a potted tomato plant each, where *B. bassiana* was inoculated in the soil and plants were subsequently sprayed with *B. cinerea*; the remaining 4 cuvettes (soil) hosted one pot each containing only soil (background cuvettes).

Absolute CO_2_ and H_2_O concentrations of the inlet and the outlet of the plant cuvettes were measured by two Infra-Red Gas Analysers (IRGA; LI-840A, LI-COR Biosciences, Lincoln, Nebraska) continuously during the entire experiment. For each measurement, gas-exchange was measured every 5 min and 20 s before switching to the next cuvette. After a 60-s purge time (to be sure that no contamination of gases from the previously measured cuvette occurred), gas exchange data were recorded. Calculations of Pn and evapo-transpiration (E-Tr) were done in Matlab vers.2017a (Mathworks Inc. Natick, Massachusetts) using the formulas given above (Von Caemmerer and Farquhar, 1981). For Pn, data from background cuvettes (containing only a pot of soil) were subtracted from plant cuvette data to account for soil contribution to CO_2_ exchange. For E-Tr, no such background correction was made, and the data also included soil evaporation. In fact, it was not possible to maintain the same soil moisture in all pots, and background corrections would give a misleading result in terms of E-Tr. In both cases, the rates were normalized for the leaf area of the plant inside the cuvette, which was estimated as discussed by Hartmann et al. (2011). Plant pictures were taken from two different angles (nine from the front view, nine from a 45^°^ angle from above) in a photo-station equipped with a turntable in which a stepper motor allowed to rotate the plants in front of an adequate image background. These measurements were done before placing the plants into the cuvettes and repeated after one week, in order to capture plant growth (Jud et al., 2018). All pixels of the images representing plant leaves were calculated and interpolated with leaf area values using a cubic spline (measured, exact leaf area vs. extracted pixels). The gas-exchange data were elaborated with the R (Vers.4.2.0, using R studio) software.

VOC analysis was performed using gas chromatography mass spectrometry (GC-MS), following established procedures (Ghirardo et al., 2020). Samples were collected from the air exiting the cuvettes in GC-MS glass tubes containing 40 mg of Tenax TA and 40 mg of Carbopack X by diverting a constant air flow of 70 mL min^-1^ through the tubes for 360 min, from 9:30 am to 3:30 pm. Sampling was conducted at 3 different times after the plants were enclosed in the VOC-SCREEN cuvettes: at 10, 12 and 15 dpi as illustrated in **Figure 1**. Each cartridge contained 859.3 pmol of δ-2-carene as internal standard. Quantification was accomplished using three calibration curves, which were generated independently in triplicate and preparing six different concentrations of pure standard mixtures (α-pinene, sabinene, limonene, methyl-salicylate, bornyl acetate, ß-caryophyllene, α-humulene). Volatiles that were not available as standards were quantified using calculated response factors leading to a quantification uncertainty of 1-8% (Ghirardo et al., 2020). Plant VOC emissions were corrected using measurements of the background cuvettes. Non-isothermal Kovats retention indices (RIs) were calculated based on chromatography retention times of a saturated alkane mixture standard (van Den Dool and Kratz, 1963). Limit of detection (LOD) was calculated using two standard deviations.

### 2.7 Statistical analysis

Data are shown as means ± standard error of means (SEM) and were subjected to analysis of variance (ANOVA) or Student’s t-test performed using the R (Vers.4.2.0, using R studio) software. To separate means within each parameter, the Tukey’s test was performed. Statistically significant differences were tested at p < 0.05.

## 3 Results

### 3.1 Colonization of tomato plants by *Beauveria bassiana* and impact on plant growth and photosynthetic gas exchange

#### 3.1.1 *Beauveria bassiana* endophytic colonization data

**Figure 2:**
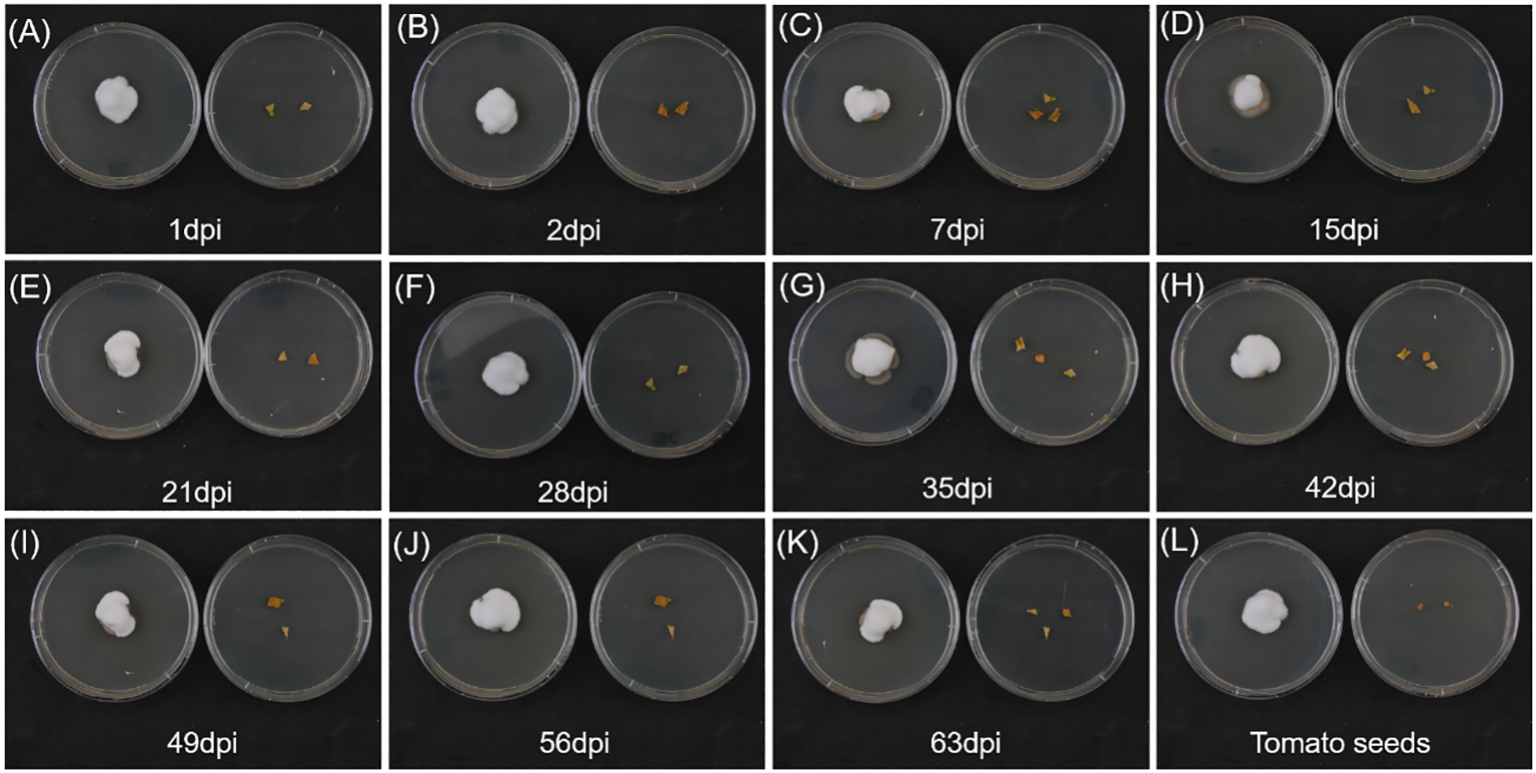
Endophytic colonization by *Beauveria bassiana* evaluated on PDA plates on leaves at 1, 2, 7, 15, 21, 28, 35, 42, 49, 56, 63 dpi (**A–K**, respectively), and on fruit seeds at 77 dpi **(L)**. Leaves or tomato seeds of *B. bassiana*-colonized plants (left plate of each panel) are compared with leaves or tomato seeds of control plants (right plate of each panel).

The presence of white, cottony, dense hyphal growth emerging from different plant-treated tissues (**Figure 2**, left side of each plate) was confirmed to be *B. bassiana* mycelium. *Beauveria bassiana* was found in tomato leaves after the second root inoculation (one dpi), and rapidly colonized all plant tissues, also being retrieved in seeds of fruits of colonized tomato plants (**Figure 2**, bottom right plate). In control plants (**Figure 2**, right side of each plate) no hyphal growth of *B. bassiana* occurred, ensuring absence of fungal contamination during the experiments.

#### 3.1.2 Plant growth data

**Figure 3:**
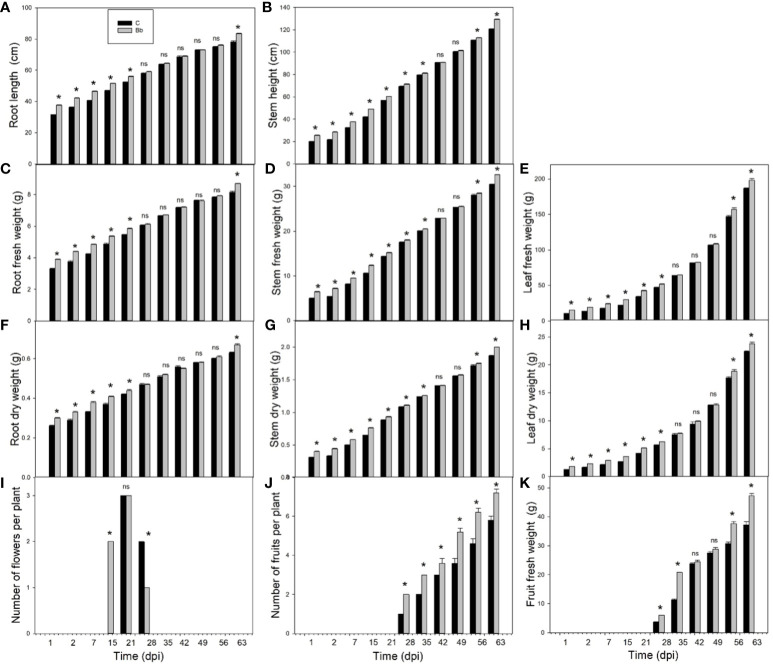
Growth data in control (C, black bars) tomato plants and in plants colonized by *Beauveria bassiana* (Bb, grey bars). Root length **(A)**, stem height **(B)**, root, stem and leaf fresh weight **(C–E)**, respectively, and dry weight **(F–H)**, respectively, numbers of flowers **(I)** and fruits **(J)** per plant, and fruit fresh weight per plant **(K)** at 1, 2, 7, 15, 21, 28, 35, 42, 49, 56, and 63 dpi. Means ± SEM (N=5) are shown. Statistical significance of differences between C and Bb means was assessed at each dpi by Student’s t-test, asterisks represent p < 0.05; ns = non-significant differences.

Colonization of *B. bassiana* led to significant (p < 0.05) increase of plant growth in terms of root length (**Figure 3A**), fresh (**Figure 3C**) and dry weight (**Figure 3F**), from the beginning of the experiment to 28 dpi; at this timepoint growth became similar in control and treated plants and then *B. bassiana*-colonized plants resumed growing more than controls at 63 dpi. Stem height (**Figure 3B**), fresh (**Figure 3D**), and dry weight (**Figure 3G**), were all promoted by *B. bassiana*-treatment with respect to controls until 35 dpi, and after 56 dpi. The same profile was followed by leaf growth, based on either fresh (**Figure 3E**) and dry weight (**Figure 3H**). Flowering started around 15 dpi in *B. bassiana*-colonized plants (**Figure 3I**). This was earlier than in controls, and late flowering was reduced in *B. bassiana*-colonized plants with respect to controls. Fruiting was also different in plants with *B. bassiana*, as more fruits where already set at 28 dpi (**Figure 3J**), and fruit-set remained significantly higher than in controls during the entire fruiting period. Tomato fruits from *B. bassiana*-colonized plants were heavier than those from control plants (**Figure 3K**).

#### 3.1.3 Gas exchange data at leaf level

**Figure 4:**
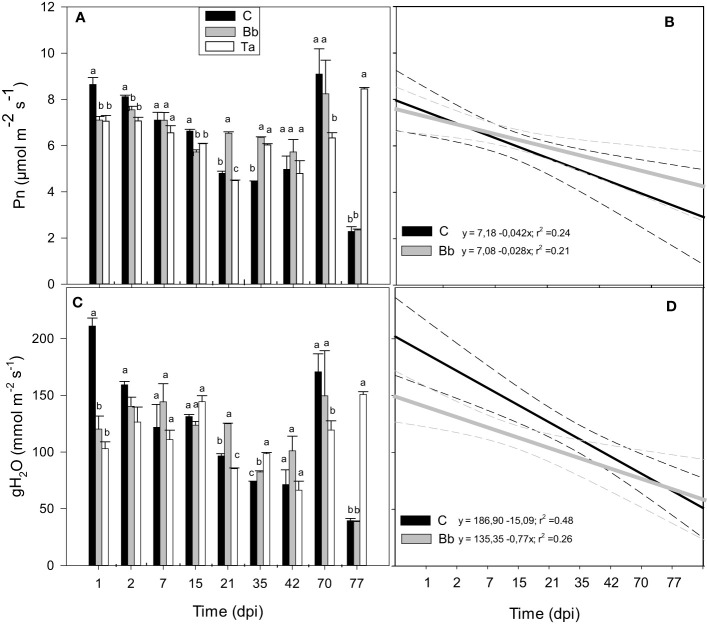
Net photosynthesis (Pn, **A**) and stomatal conductance (gH_2_O, **C**) of leaves of control tomato plants (C), and of plants colonized by *Beauveria bassiana* (Bb), and *Trichoderma afroharzianum* (Ta) at 1, 2, 7, 15, 21, 35, 42, 70, 77 dpi during the first experiment in Italy. Statistical significance of differences among the means of the different treatments was assessed over single time point, as indicated by the dashed lines, by ANOVA followed by Tukey’s test. Means ± SEM (N=3) are shown. Different letters indicate significantly different means with p < 0.05. In **(B, D)**, linear regression lines for Pn (black) and gH_2_O (grey) over time (dpi) are shown for control tomato plants and plants colonized by *B. bassiana*. The 95% confidence interval with respect to best fit lines is represented by the dashed lines of the same color of the regression lines.

During the first year experiment the impact on photosynthetic gas exchange of *B. bassiana* was compared with that of the well-known biocontrol agent and growth inducer *Trichoderma afroharzianum* (Thapa et al., 2020). The maximal quantum yield of PSII (Fv/Fm) did not change among controls and plants inoculated with *B. bassiana* or *T. afroharzianum*, along the time-course of the experiment (**Supplementary Figure S1**), respectively. However, both *B. bassiana* and *T. afroharzianum* induced a significant but transient (one to 2 dpi-long) reduction of net photosynthesis (Pn) and stomatal conductance (gH_2_O) (**Figures 4A, B**). At 21 and 35 dpi, *B. bassiana* improved Pn of tomato plants with respect to controls (p < 0.05), but at 42 dpi Pn and gH_2_O, were not different in all conditions. Measurements made 70 and 77 dpi rendered erratic results on the few plants still alive (**Figure 4B**). Aging was faster in control plants than in plants colonized by *B. bassiana* or *T. afroharzianum*, as shown by the steeper regression line fitted to the data of Pn and gH_2_O (**Figures 4C, D**).

**Figure 5:**
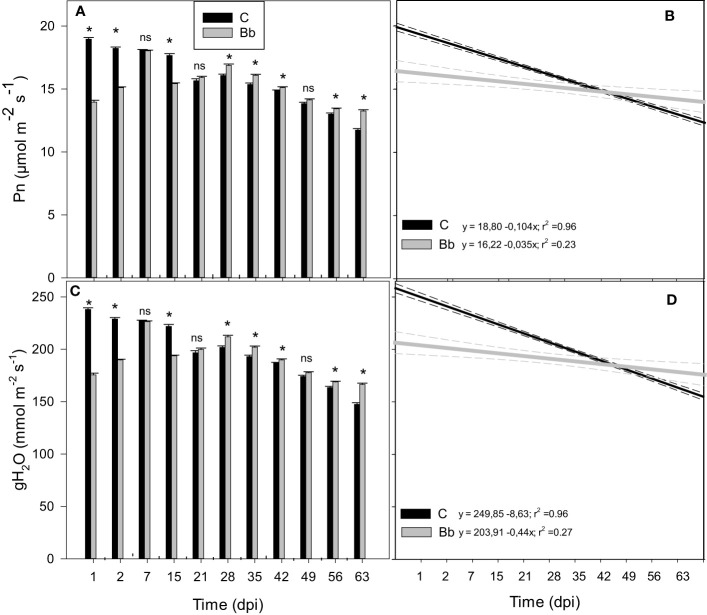
Net photosynthesis (Pn, **A**) and stomatal conductance (gH_2_O, **C**) of leaves of control tomato plants (C), and of plants colonized by *Beauveria bassiana* (Bb) at 1, 2, 7, 15, 21, 28, 35, 42, 49, 56, 63 dpi, measured during the second experiment in Germany. Statistical significance of differences among the means of the different treatments was assessed over single time point, as indicated by the dashed lines, by ANOVA followed by Tukey’s test. Means ± SEM (N=3) are shown. Asterisks and ns indicate significantly and non-significantly different means with p < 0.05, respectively. In **(B, D)**, linear regression lines for Pn (black) and gH_2_O (grey) over time (dpi) are shown for control tomato plants and plants colonized by *B. bassiana*. The 95% confidence interval with respect to best fit lines is represented by the dashed lines of the same color of the regression lines.

Gas-exchange measurements at leaf level were repeated during the second experiment at HMGU. Results of the first experiment could be largely confirmed by the second experiment: *B. bassiana* application induced a rapid and significant reduction of Pn and gH_2_O compared to control plants within the first days after root inoculation (**Figures 5A, B**). This reduction was visible until two dpi, after which gas-exchange parameters of plants colonized by *B. bassiana* resumed the levels observed in controls. From 28 dpi on, however, Pn and gH_2_O became higher in the plants treated with *B. bassiana* compared to controls. Also in this second experiment, Pn and gH_2_O decreased over time both in controls and in plants treated with *B. bassiana*, and aging was faster in control plants, as shown by the different slopes of **Figures 5C, D**.

### 3.2 Impact of colonization of tomato plants by *Beauveria bassiana* on the development of *Botrytis cinerea* infection

**Figure 6:**
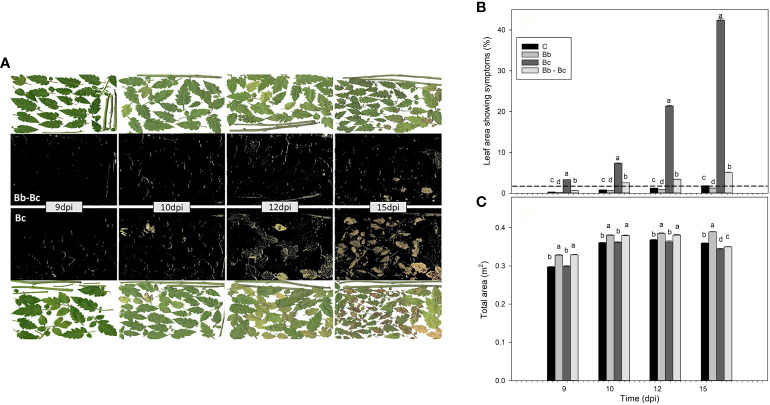
Evaluation of *Botrytis cinerea* infection in plants without (Bc) or with *Beauveria bassiana* colonization (Bb-Bc) at 9, 10, 12, 15 dpi (see **Figure 1A** for experimental design), as compared to controls (C) and to plants treated with *B. bassiana* (Bb). In **(A)**, the digital RGB images of scanned green leaves of Bc (lower plates) and Bb-Bc plants (upper plates) are shown on a white background. The black background images were obtained with the leaf area calculator software, to better identify the damage caused by *B. cinerea* infection. In **(B)**, the percentage of leaf area showing symptoms with respect to the total area of plants **(C)** is shown. In **(B)** the symptoms attributed to C and Bb plants (below the dashed line) are not related to damage caused by *B. cinerea* infection, rather representing chlorotic spots due to aging or other non-pathological causes. In both **(B, C)**, statistical significance of differences among the means of the four treatments was assessed at each dpi by ANOVA followed by Tukey’s test. Means ± SEM (N=5) are shown, and different letters indicate statistically different means with p < 0.05.

Plants colonized by *B. bassiana* were less damaged than controls when exposed to *B. cinerea* infection. The effect was barely noticeable with a visual inspection at 9 dpi but became increasingly evident with time (**Figure 6A**). By using a calculation software, we were able to translate this visual effect into measurable data (**Figure 6B**). At 15 dpi more than 40% and less than 5% of the foliar surface area was damaged by *B. cinerea* in controls and *B. bassiana*-colonized plants, respectively (**Figure 6B**). Chlorotic and necrotic symptoms could both be seen in the RGB images of severely infected *B. cinerea* leaves, but to a much lower extent chlorotic spots also appeared on control plants and *B. bassiana*-colonized plants (**Figure 6B**). These symptoms are not related to *B. cinerea* infection, rather often revealing initial leaf ageing. These chlorotic areas are visualized to give a complete picture of the results but will be then subtracted to the total biomass of controls and *B. bassiana*-colonized plants to correctly interpret the damage specifically caused by *B. cinerea* infection. This experiment also confirmed that plants treated with *B. bassiana* grew more than control plants (**Figure 6C**), as the increase in total area is comparable to the increase of leaf weight observed in **Figures 3E, H**.

### 3.3 Impact of colonization by *Beauveria bassiana* and of *Botrytis cinerea* infection on gas-exchange and VOC emission of whole tomato plants

#### 3.3.1 Gas-exchange at entire plant level

The antagonistic effect of *B. bassiana* on *B. cinerea* infection was further evaluated by analysis of gas exchange and VOC emission from entire plants. Net photosynthesis (Pn), dark respiration (Rd), and evapo-transpiration (E-Tr) were monitored continuously (24-h long) from 9 dpi until 16 dpi (see **Figure 1A** for the complete experimental design). Measurements on the first and the last day (when plants were inserted into and removed from the cuvettes) were not considered in our analysis, and only data from 10 until 15 dpi are shown in **Supplementary Figure S2**.

**Figure 7:**
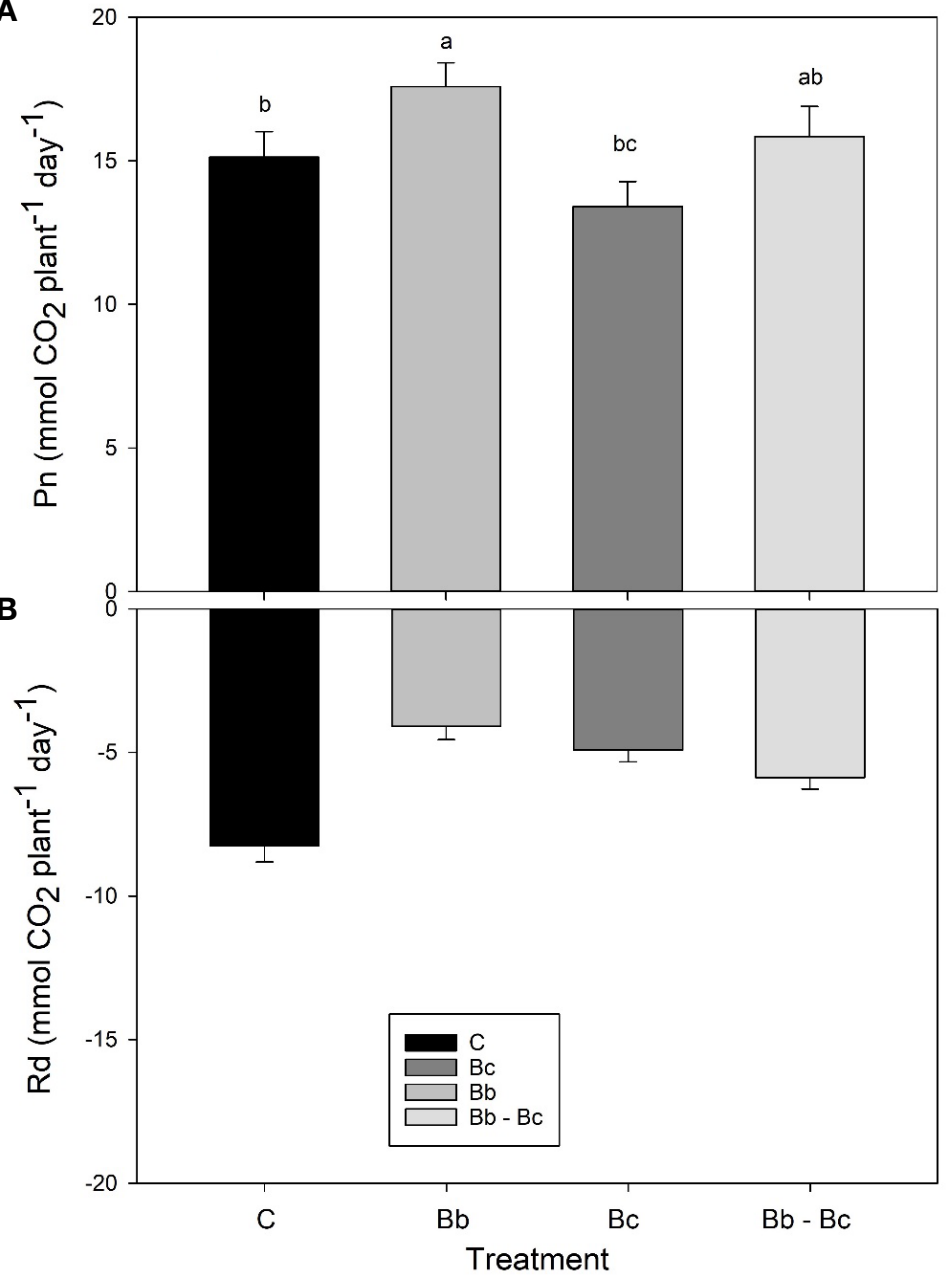
Net photosynthesis (Pn, **A**) and dark respiration (Rd, **B**) from whole plants of tomato in the VOC-SCREEN platform. Control plants (C) are compared with plants treated with *Beauveria bassiana* (Bb), *Botrytis cinerea* (Bc), and *B. bassiana* and *B. cinerea* (Bb-Bc). Values are represented for each treatment as an average of all days **(A)** or nights **(B)** of experiment. Statistical significance of differences among the four treatments was assessed over the entire experimental period by one-way ANOVA followed by Tukey’s test. Means ± SEM (N=5) are presented, and statistically different means (p < 0.05) are shown with different letters.

Based on results shown in **Supplementary Figure S2**, data were further filtered and averaged on two periods: h 8:00-15:00 (when PAR in cuvettes was stable around 700 μmol m^-2^ s^-1^); and h 20:00-4:00 (when PAR was turned off). In these two periods Pn (in the light) and Rd (in the dark) were relatively constant and could be integrated on whole plant and whole day basis. Plants colonized by *B. bassiana* showed significantly higher Pn and lower Rd than all other plants. On the other hand, plants infected by *B. cinerea* that significantly reduced the photosynthetic areas (**Figure 6C**) showed the lowest Pn (**Figure 7**).

**Figure 8:**
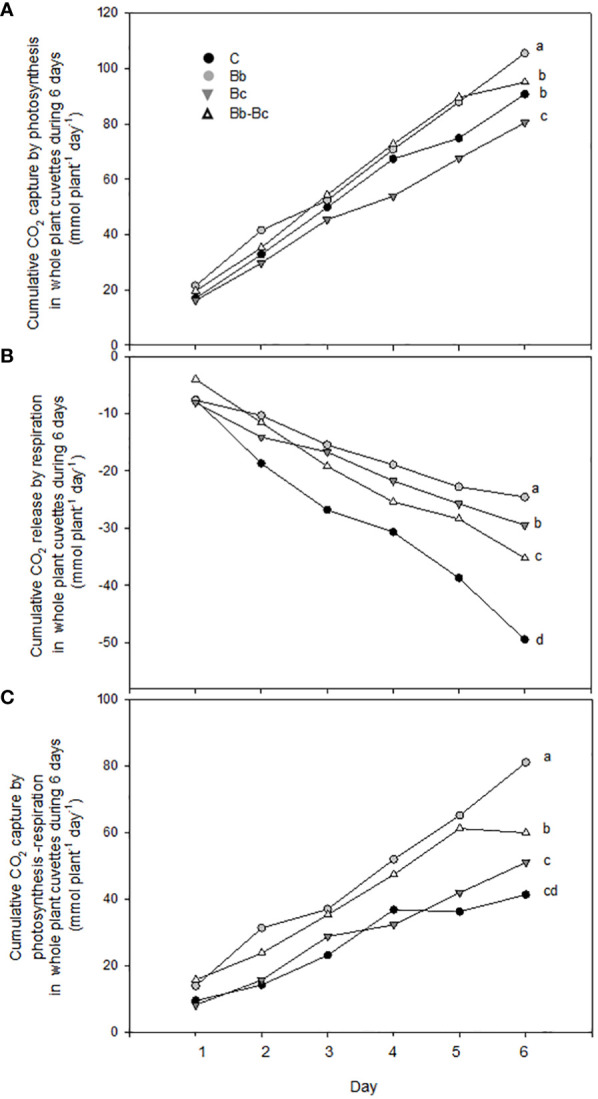
Cumulative CO_2_ capture by photosynthesis **(A)**, release by respiration **(B)** and net capture by photosynthesis-respiration **(C)** in whole plant cuvettes (N = 5 per treatment) are shown summing up parameters measured each day during a 6-day long period, starting at 9 dpi (as shown in the text). Control plants C are compared with plants treated with *Beauveria bassiana* (Bb), *Botrytis cinerea* (Bc), and *B. bassiana* and *B. cinerea* (Bb-Bc). Max SEM among the 5 cuvettes per treatment and per day was always less than 3% of the reported data. Statistical significance of differences among the four treatments was assessed on the cumulated data (last data point) by one-way ANOVA followed by Tukey’s test. Statistically different means (p < 0.05) are shown with different letters.

When examining the cumulative exchange of CO_2_ by the plants over the entire experimental period (six days), *B. bassiana*-colonized plants and controls photosynthesized slightly more than plants infected by *B. cinerea* and those that were infected by *B. cinerea* and colonized by *B. bassiana* (**Figure 8A**). However, Rd measurements in the night confirmed a much higher respiratory emission of CO_2_ by control plants (**Figure 8B**), which reduced the total uptake of CO_2_ (net photosynthesis – respiration) in comparison to plants colonized by *B. bassiana* (**Figure 8C**).

#### 3.3.2 VOC emissions at whole-plant level

**Figure 9:**
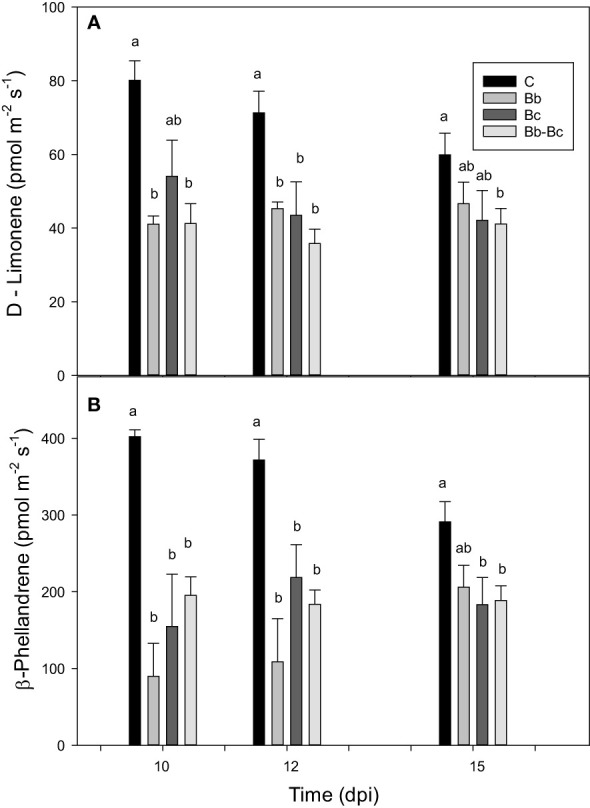
Comparison of the main two VOCs (D-limonene **(A)** and β-phellandrene **(B)**) emitted by control tomato plants (C), and by plants treated with *Beauveria bassiana* (Bb), *Botrytis cinerea* (Bc), and both *B. bassiana* and *B. cinerea* (Bb-Bc) at 10, 12 and 15 dpi. Statistical significance of differences among the means of the four treatments was assessed at each dpi by one-way ANOVA followed by Tukey’s test. Means ± SEM (N=5) are shown, and statistically significant means (p < 0.05) are separated with different letters.

We investigated the changes in tomato VOC emissions consequent to *B. bassiana* colonization. Different classes of VOCs were identified according to library match and Kovats's RIs. However, the profile of emitted VOCs was similar under all treatments. Main VOCs emitted were the monoterpenes β-phellandrene, α-pinene, p-cymene, D-limonene; the sesquiterpene β‐caryophyllene; the benzene analogues m-xylene and phenylethyne (the latter tentatively identified); the ethanol ester triethyl phosphate (possibly a contaminant); and the saturated fatty aldehydes decanal and nonanal (**Supplementary Table S1**). β-Phellandrene, and D-limonene were the two most emitted VOCs, with β-phellandrene contributing on average to around 75% of the total emissions in all treatments (**Figure 9**). Treatments with *B. bassiana* and *B. cinerea* (alone or in combination) did not elicit the emission of induced VOCs, rather they reduced the overall constitutive emission of VOCs. A general but not statistically significant reduction of VOC emission from the first to the third sampling point (in leaves 44 to 50 d-old) was also observed, particularly in control plants (**Figure 9**).

## 4 Discussion

Beneficial associations between plants and microorganisms have been extensively studied (Yan et al., 2019; Adeleke and Babalola, 2022; Dlamini et al., 2022; Russo et al., 2022) and are often linked to plant physiological and metabolic reprogramming that promote growth and strengthen the defense barriers (De Palma et al., 2019). Recently, the communication mechanisms between plants and endophytic fungi in presence of biotic and abiotic stresses was deeply investigated (Lu et al., 2021), and some fungal entomopathogens started to be proposed as beneficial endophytes (Vidal and Jaber, 2015; Jaber and Enkerli, 2017). With this work we intended to test whether the entomopathogenic fungus *B. bassiana*: a) successfully colonizes the different organs of tomato; b) is perceived as a stressful or beneficial organism during different stages of plant development with an overall positive effect on plant phenotypes; c) defends tomato plant against the pathogenic fungus *B. cinerea*.

Colonization by *B. bassiana* of internal plant tissues has been observed in many crops (Vega, 2018; Mantzoukas et al., 2021; Mantzoukas et al., 2022), including tomato (Proietti et al., 2023). As the inoculum source was in the soil (we irrigated the plants with a solution of spores), *B. bassiana* could have entered the plant via the root system, or could have travelled out of the plant reaching stem lenticels close to the soil surface from where invaded the plant. *Beauveria bassiana* colonization was successfully verified in the leaves that were used for gas-exchange analyses. The infection was extremely rapid (within one dpi), and the leaves were proved to be colonized at all tested timepoints. First vertical transmission such as shown here was proved by Quesada-Moraga et al. (2014) in *Papaver somniferum*. We show for the first time that *B. bassiana* colonization reach tomato fruits and seeds, making trans-generation transmission also possible. If from a biological control perspective this could represent an advantage (allowing protection to different generations), fruit colonization by *B. bassiana* also makes us wonder if tomatoes of plants treated with this beneficial microorganism can be eaten safely. *Beauveria bassiana* produces a mycotoxin (beauvericin) with insecticidal activity, which is the basis of *B. bassiana* entomo-pathogenicity (Al Khoury et al., 2020). When produced by *Fusarium* sp. beauvericin may be toxic also to mammals (Logrieco et al., 1998). More studies are needed to test whether *B. bassiana* treatments may impair consumption of tomatoes.

Plants responded fast to *B. bassiana* colonization. We showed that the infection was extremely rapid. The photochemistry of photosynthesis was clearly unaffected by *B. bassiana* infection, as shown by the steady-state values of the maximal quantum yield of PSII (Fv/Fm). However, both *B. bassiana* and *T. afroharzianum* induced a significant but transient (1-2 day-long) reduction of stomatal conductance and net photosynthesis. Our results suggest the induction of a rapid activation of defensive (rejection) responses against a foreign organism that invades plant organs. This might have primed a plant response that should be further demonstrated by examining activation of defensive metabolism (e.g., priming of antioxidant metabolites) (Pollastri et al., 2021). Fast perturbations of transcriptomes of tomatoes after *T. afroharzianum* root treatment were previous detected (De Palma et al., 2019). ROS signalling, SA responses and cell wall modifications were activated 24 h after treating the plants, whereas after 72 h an increased transcription of ethylene and auxin signalling genes triggered possible modifications in root architecture, and possibly also plant growth stimulation. As discussed elsewhere (De Palma et al., 2019; Proietti et al., 2023) a down-regulation of proteins related to defense responses and up-regulation of proteins related to calcium transport during early phases of *B. bassiana* colonization of tomato plants was showed. The data set discussed by Proietti et al. (2023) is delayed by 3 days with respect to our very rapid reduction of Pn and gH_2_O and may reflect the following establishment of a symbiotic relationship. For example, calcium flux across the plasma membrane was found to be an early signalling step when establishing symbiosis and immunity (Yuan et al., 2017; Moscatiello et al., 2018; Jiang and Ding, 2022).

We did not notice any significant and prolonged stimulation of leaf photosynthesis, implying that growth stimulation may not be due to an improvement of carbon fixation on a leaf area unit. However, slower plant aging might have been related to delayed reduction in Pn and gH_2_O in plants colonized by *B. bassiana* with respect to control plants. Moreover, a significant effect of enhanced growth was seen when measuring net photosynthesis at whole plant level rather than after normalizing on a leaf area basis. Finally, respiratory losses of carbon overnight (Rd) were also reduced in plants colonized by *B. bassiana* with respect to all other treatments, which may also support better carbon availability/allocation for growth and development. However, a direct correlation between net photosynthesis and dark respiration (i.e. a simultaneous increase of Pn and Rd) is more often observed (Poorter and Bongers, 2006). The lower respiration rate found in our study may rather confirm slower ageing and prolonged leaf life-span (Reich et al., 1998) in *B. bassiana*-treated plants than in controls that do not interact with the BM. Interestingly, our results indicate that any interaction with fungi (either pathogenic or beneficial) has a significant inhibitory effect on dark respiration in tomato.

In other crops, like corn, grapevine, bean, cotton, coffee, and sorghum, *B. bassiana* endophytic colonization was proposed to result in plant growth promotion (Posada et al., 2007; Tefera and Vidal, 2009; Lopez and Sword, 2015; Ramos et al., 2017; Afandhi et al., 2019; Russo et al., 2019; Mantzoukas et al., 2021). This was also the case with tomato. Colonization of *B. bassiana* led to statistically significant increase of all plant organs (roots, stems and leaves). We observed that growth stimulation was stronger early after the infection, was absent or somehow reduced in the second month after the infection, and then again became evident on older plants. This confirms recently published data showing a reprogramming of the proteome toward energy production processes sustaining growth in ageing plants (Proietti et al., 2023). We note that both fresh and dry weights of the phenotyped plant parts increased, suggesting that the effect was not simply limited to an improved water content of the plants.

This is to our knowledge the first experiment that has followed the interaction between tomato and *B. bassiana* along the entire plant life. The infection of *B. bassiana* produced an unexpected anticipation of flowering, and a positive effect on tomato fruit-set. Not only more fruits were set during the entire fruiting period, but fruits were also significantly bigger in *B. bassiana-*treated plants than in controls. This result confirms that *B. bassiana* may be used as a growth stimulator of tomato plants, as suggested earlier in a different experiment on the basis of biochemical responses (Proietti et al., 2023). Normally, BMs promote plant growth either by directly facilitating nutrient uptake (as biofertilizers) or by modulating (stimulating) plant hormone levels (Russo et al., 2022). Improved nutrient uptake is frequent in the case of soil BMs that do not act as endophytes (Nasslahsen et al., 2022), although it was reported that *B. bassiana* might enhance nutrient availability, particularly soluble phosphate (Barra-Bucarei et al., 2019a). Endophytization is more likely to activate plant hormones, especially those involved in plant growth and development (Waadt et al., 2022). In particular, the role of gibberellins (GAs) in the development and maintenance of plant-beneficial microbe symbioses is an emerging area of research (McGuiness et al., 2019). A significant up-regulation of growth-related hormones like GA precursors and their active forms was observed in *B. bassiana*-treated tomato plants, together with an increased biosynthesis of hormones related to defense such as benzoic acid and jasmonate (Proietti et al., 2023). Entomopathogenic fungi control pests (Mantzoukas et al., 2015; Mantzoukas and Lagogiannis, 2019; Bava et al., 2022). However, several studies have demonstrated that endophytic fungi can also protect host plants against pathogens (Ownley et al., 2008; Barra-Bucarei et al., 2019b) and herbivores (Arnold et al., 2003; Ownley et al., 2004; Card et al., 2016; Jensen et al., 2020). Recent results have suggested an antifungal activity of *B. bassiana* against *B. cinerea* (Barra-Bucarei et al., 2020; Sinno et al., 2021; Proietti et al., 2023). *Botrytis cinerea* is a necrotrophic fungus. It first produces toxic compounds that cause cell death, and then the fungus feeds on the dead tissue, causing typical necrotic lesions (Williamson et al., 2007). We were able to monitor *B. bassiana* protection of tomato plants against *B. cinerea* during a long period (until the symptoms of the pathogen made it impossible to continue with the analysis), and to visually see its protective effect on the entire plant. *Beauveria bassiana* reduced *B. cinerea* symptoms in leaves almost entirely. As previously described (Ownley et al., 2004; Ownley et al., 2008), the endophyte might have an indirect effect if it moves through the vascular system until it reaches the leaf tissues, and competes for space and food with the pathogen, reducing its colonizing ability; or it could directly parasitize the pathogen (mycoparasitism), weakening its pathogenic potential. *Beauveria bassiana* may also have a role in reducing the oxidative potential, which often is a factor inducing *B. cinerea* infection (Govrin and Levine, 2000; Kuźniak and Skłodowska, 2004). A reduction of ROS and of lipid peroxidation indicators was observed in leaves colonized by *B. bassiana* and infected by *B. cinerea*, with respect to plants only infected with *B. cinerea* (Proietti et al., 2023). However, the mechanism underlying improved plant protection against *B. cinerea* in plants colonized by *B. bassiana* should be further elucidated with dedicated experiments.

We further examined VOCs to assess whether colonization by the entomopathogen *B. bassiana* aids plants fight *B. cinerea* infection through fungi-induced plant VOC emissions or potentially via direct emissions of volatiles from *B. bassiana.* Unexpectedly, we did not observe any enhanced or induced emissions in the bouquet of volatiles from tomato plants colonized by *B. bassiana*. VOCs can mediate plant-plant communication (Rosenkranz et al., 2021) and BMs can also alter plant VOC profile by interaction (Russo et al., 2022). While plant VOCs hold promise as a natural and eco-friendly solution to defend plants from biotic stresses (Brilli et al., 2019), their effectiveness remain uncertain. In the case of grapevine, *B. bassiana* elicited VOC emissions, although this induction did not result in improved insect resistance (Moloinyane and Nchu, 2019). Tomato plants primarily store terpenes in glandular trichomes of leaves and stems (Catola et al., 2018), which are filled during the early stage of leaf development. The glandular VOCs are either released in large amounts upon rupture of the cuticle (stress inductions) or slowly evaporate out of glands (constitutive releases). Burst of volatiles are commonly observed in plant species that possess specialized storage structures such as secretory cavities, resin ducts and glandular trichomes when attacked by insects or because of generic mechanical stresses (Loreto et al., 2000; Kang et al., 2010) and rupture of glandular trichomes can induce the expression of defense-related genes in tomato plants (Peiffer et al., 2009). Contrary to expectations, we did not detect a burst of volatile emissions suggesting that the growth of the fungi was not sufficient to damage the tomato glands, which would have stimulated glandular-dependent defense responses. We neither detected stress-induced emissions, at least in the time frame we sampled (4 days after *B. cinerea* leaf infection and 10 days after root inoculation with *B. bassiana*). In contrast, constitutive plant VOC emissions significantly decreased in *B. bassiana*-colonized leaves, as well as in leaves treated with *B. cinerea* and both fungi together. Decrease of plant VOC emissions after interaction with BMs was observed in mycorrhized beans (Babikova et al., 2014). One possible explanation for the reduction of the total VOC emissions could be a depletion of the terpene pool resulting from the interaction with the fungi.

Overall, we interpret our VOC results as an indication that improved protection against *B. cinerea* by *B. bassiana* does not involve VOC signalling. Nevertheless, our measurements showed a relevant decrease (up to 80% in early phases of colonization/infestation) of β-phellandrene and D-limonene, the two major contributors (<90%) commonly found in the tomato volatile bouquet (Jansen et al., 2009). Notably, β-phellandrene is known to attract natural enemies (Colazza et al., 2004; Rasman et al., 2005), hence the observed decrease in emission capacities upon *B. bassiana* colonization could have significant ecological consequences in tritrophic interactions.

The reduced total VOC emission might have been caused by the fact that in plants with long-term endophytization by *B. bassiana* less stress-signalling compounds are induced, or that emitted plant VOCs are absorbed by the endophytic fungus before emission. Further studies are needed to explain this result. Reduced VOC emission might also contribute to save carbon and energy for sustained growth and development of plants colonized by *B. bassiana*.

It should also be noted that unfortunately we did not measure VOCs when *B. bassiana* was temporarily rejected by plants, as indicated by the reduction of net photosynthesis and stomatal conductance (1-2 dpi) in both of our experiments. This stimulation might have been associated to a temporary rise of VOCs that might serve to prime defenses (Pollastri et al., 2021). A rapid induction of monoterpenes was indeed reported in tomato plants infected by *B. cinerea* (Jansen et al., 2009). This should be assessed with future experiments, as it might be essential to fully understand the impact of *B. bassiana* in tomato leaves, especially if combined with detection of ROS and defensive metabolites in colonized leaves (Stamelou et al., 2021).

## 5 Conclusions

Drawing on our results, we suggest that tomato plants in the beginning perceive treatments with BMs (such as *B. bassiana* and *T. afroharzianum*) as an ‘infection’, translated into a significant but short-term transient reduction in stomatal conductance and net photosynthesis, and a possible and transient priming of defensive metabolites. Thereafter, *B. bassiana* appears to establish itself as an endophyte in tomatoes, stimulating plant growth and productivity. Perhaps even more interestingly, *B. bassiana* seems to control the infection of the widespread pathogen *B. cinerea*, largely reducing the negative symptoms at foliar level. VOC emissions did not explain how *B. bassiana* controlled the pathogen, but VOC reduction might be interpreted as mirroring an improved plant health status. These findings expand the possible use of *B. bassiana* from being employed as an entomopathogen to a general and promising use as a plant growth promoter and defender. Further studies should focus on the mechanisms driving first negative (lower photosynthesis and stomatal conductance) and then positive (higher photosynthesis and growth, lower respiration) plant responses to *B. bassiana*, and should also enquire whether such responses are widespread and durable in other crops and in natural vegetation.

## Data availability statement

The raw data supporting the conclusions of this article will be made available by the authors, without undue reservation.

## Author contributions

AR: Investigation, Data curation, Methodology, Writing – original draft. JW: Data curation, Investigation, Methodology, Writing – review & editing. AG: Data curation, Investigation, Methodology, Writing – review & editing, Validation. SP: Data curation, Investigation, Methodology, Writing – review & editing. MM: Data curation, Investigation, Methodology, Writing – review & editing. MR: Data curation, Investigation, Methodology, Writing – review & editing. JS: Writing – review & editing, Conceptualization, Validation, Visualization. FL: Conceptualization, Validation, Visualization, Writing – review & editing, Funding acquisition, Investigation, Resources, Supervision.
