# Supplementary material for: Interaction with the entomopathogenic fungus Beauveria bassiana influences tomato phenome and promotes resistance to Botrytis cinerea infection
Source: Front Plant Sci. 2023 Dec 19;14:1309747. doi: 10.3389/fpls.2023.1309747 (PMC10762804; doi:10.3389/fpls.2023.1309747)
Supplement: Supplementary file 1 [file DataSheet_1.docx]

Interaction with the entomopathogenic fungus *Beauveria bassiana* influences tomato phenome and promotes resistance to *Botrytis cinerea* infection

Assunta Russo^1,3^, Jana Barbro Winkler^2^, Andrea Ghirardo^2^, Maurilia M. Monti^3^, Susanna Pollastri^3^, Michelina Ruocco^3^, Jörg-Peter Schnitzler^2*^, Francesco Loreto^3, 4*^

Supplementary Material

# Supplementary Figures and Tables

## Supplementary Figures

**
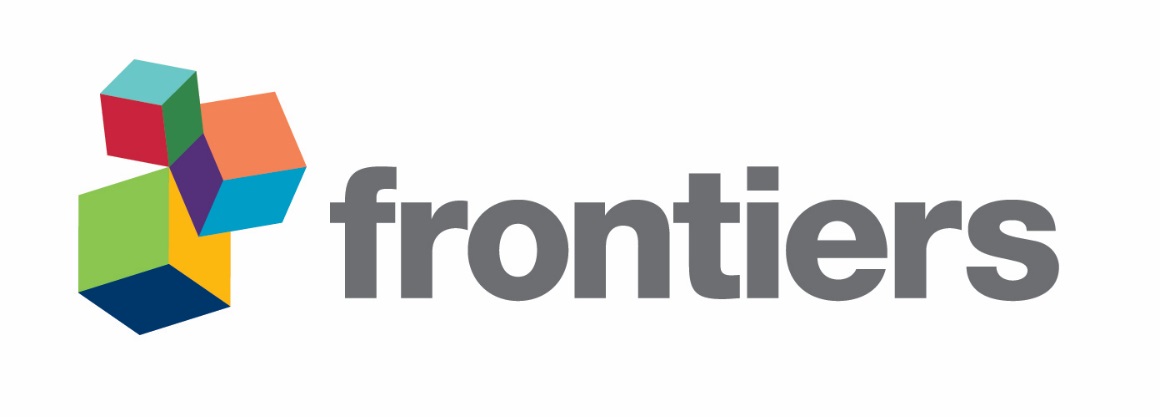
**

**Supplementary Figure S1.** Maximal quantum yield of PSII (Fv/Fm) of tomato control plants, or plants colonized by *B. bassiana*, or *T. afroharzianum* after 1, 2, 7, 15, 21, 35, 42, 70, 77 dpi. Means ± SEM (N=5) are shown. Statistical significance of differences among the means was assessed over the entire experimental period by ANOVA followed by Tukey's test, p < 0.05. No statistically significant difference among treatments was observed.

**Supplementary Figure S2.** Measurements of daily profiles of net photosynthesis and evapo-transpiration collected from whole plants in real-time from 10 dpi to 15 dpi in controls (C) and in plants treated with *B. bassiana* (Bb), *B.cinerea* (Bc) and *B. bassiana and B. cinerea* (Bb-Bc). Data are presented after normalization, as for foliar gas exchange (mols of gases m^-2^ s^-1^). Other details are reported in the main text.


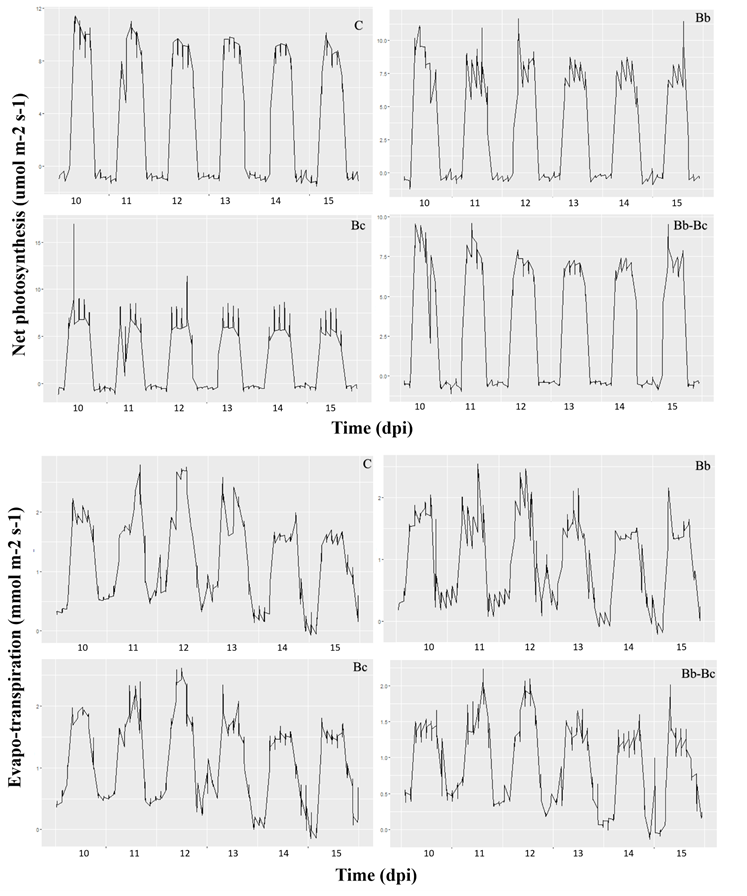


**Supplementary Table S1.** Identification of VOCs from tomato (*Solanum lycopersicum L.*, cultivar “San Marzano nano”) plants based on database matches and Kovats’ retention index (RI). RI_Exp_, experimental RI; RI_Lit_, literature RI. RI_Lit_ values are the median ± SD of available literature values (National Institute of Standards and Technology database of 2020).

| **VOC** | **RI_Exp_** | **RI_Lit_** |
| --- | --- | --- |
| Phenylethyne | 874 | 834±4 |
| m-Xylene | 892 | 866±6 |
| α-Pinene | 934 | 937±3 |
| p-Cymene | 1023 | 1025±2 |
| D-limonene | 1028 | 1031±1 |
| β-Phellandrene | 1029 | 1031±2 |
| Nonanal | 1096 | 1104±2 |
| Decanal | 1196 | 1206±2 |
| β-Caryophyllene | 1423 | 1419±3 |
